# Supplementary material for: In Silico and In Vitro Analyses of Angiotensin-I Converting Enzyme Inhibitory and Antioxidant Activities of Enzymatic Protein Hydrolysates from Taiwan Mackerel (Scomber australasicus) Steaming Juice
Source: Foods. 2022 Jun 17;11(12):1785. doi: 10.3390/foods11121785 (PMC9222390; doi:10.3390/foods11121785)
Supplement: Supplementary file 1 [file foods-11-01785-s001.zip › foods-1743229-supplementary.pdf]

In Silico and In Vitro Analyses of Angiotensin-I Converting Enzyme Inhibitory and Antioxidant Activities of Enzymatic Protein Hydrolysates from Taiwan Mackerel (*Scomber australasicus*) Steaming Juice

Fenny Crista A. Panjaitan [1], Ting-Yi Chen [2], Hao-Hsiang Ku [3] and Yu-Wei Chang [2,*]

[1] Marine Products Processing Study Program, Marine and Fisheries Polytechnic of Jembrana, Bali 82218, Indonesia; fennycap@gmail.com
[2] Department of Food Science, National Taiwan Ocean University, Keelung City 20224, Taiwan; grazychen@gmail.com
[3] Institute of Food Safety and Risk Management, National Taiwan Ocean University, Keelung City 20224, Taiwan; kuhh@email.ntou.edu.tw
* Correspondence: bweichang@mail.ntou.edu.tw; Tel.: +886-2-2462-2192 (ext. 5152)

Table S1. Protein identified from mackerel steaming juice (MSJ), MSJ and mackerel meat SDS extract by SDS-PAGE and LC-MS/MS analysis.

Band	Protein	Accession number	Score	Sequence coverage (%)	Length (AA)	Molecular (kDa) weight from database
A1 (241 kDa)	Beta-enolase OS=*Salmo salar*	B5DGQ7	154	15.2	434	47.257
	Glyceraldehyde-3-phosphate dehydrogenase OS=*Danio rerio*	Q5XJ10	48	4.5	333	35.761
	Collagen alpha-2(I) chain OS=*Oncorhynchus mykiss*	O93484	37	3.1	1356	126.908

	Fructose-bisphosphate aldolase A OS=*Salmo salar*	B5DGM7	28	3.9	363	39.531
A2 (127 kDa)	Collagen alpha-2(I) chain OS=*Oncorhynchus mykiss*	O93484	27	0.7	1356	126.908
A3 (91 kDa)	Myosin heavy chain, fast skeletal muscle OS=*Cyprinus carpio*	Q90339	164	1.8	1935	221.462
	Tropomyosin alpha-1 chain OS=*Danio rerio*	P13104	89	13.7	284	32.703
	Alpha-enolase OS=*Thunnus albacare*s	I0J1J1	76	6.7	432	47.106
	Glyceraldehyde-3-phosphate dehydrogenase OS=*Danio rerio*	Q5XJ10	42	4.5	333	35.761
A4 (70 kDa)	Myosin heavy cha in, fast skeletal muscle OS=*Cyprinus carpio*	Q90339	1432	13.6	1935	221.462
	Beta-enolase OS=Salmo salar	B5DGQ7	237	23	434	47.257
	Tropomyosin alpha-1 chain OS=*Danio rerio*	P13104	160	21.5	284	32.703
	Glyceraldehyde-3-phosphate dehydrogenase OS=*Danio rerio*	Q5XJ10	65	10.8	333	35.761
	Fructose-bisphosphate aldolase A OS=*Salmo salar*	B5DGM7	42	3.9	363	39.531
A5 (42 kDa)	Myosin heavy chain, fast skeletal muscle OS=*Cyprinus carpio*	Q90339	4609	31.9	1935	221.462

	Tropomyosin alpha-1 chain OS=*Liza aurata*	P84335	1630	71.1	284	32.710
	Tropomyosin alpha-1 chain OS=*Danio rerio*	P13104	1289	58.5	284	32.703
	Actin, alpha skeletal muscle OS=*Carassius auratus*	P49055	1088	54.6	377	41.944
	Alpha-enolase OS=*Thunnus albacares*	I0J1J1	270	15.5	432	47.106
	Glyceraldehyde-3-phosphate dehydrogenase OS=*Danio rerio*	Q5XJ10	250	17.4	333	35.761
	Beta-enolase OS=*Salmo salar*	B5DGQ7	207	29.5	434	47.257
	Fructose-bisphosphate aldolase A OS=*Salmo salar*	B5DGM7	203	12.9	363	39.531
	Collagen alpha-2(I) chain OS=*Oncorhynchus mykiss*	O93484	32	4.1	1356	126.908
	Myosin light chain 1, skeletal muscle isoform OS=*Liza ramada*	P82159	31	5.9	186	200.54
A6 (37 kDa)	Myosin heavy chain, fast skeletal muscle OS=*Cyprinus carpio*	Q90339	2795	25.3	1935	221.462
	Tropomyosin alpha-1 chain OS=*Liza aurata*	P84335	1700	76.4	284	32.710
	Tropomyosin alpha-1 chain OS=*Danio rerio*	P13104	1272	65.8	284	32.703
	Glyceraldehyde-3-phosphate dehydrogenase OS=*Danio rerio*	Q5XJ10	276	28.2	333	35.761

	Actin, alpha skeletal muscle OS=*Carassius auratus*	P49055	240	34	377	41.944
	Fructose-bisphosphate aldolase A OS=*Salmo salar*	B5DGM7	224	14.9	363	39.531
	Alpha-enolase OS=*Thunnus albacares*	I0J1J1	75	7.2	432	47.106
	Fructose-bisphosphate aldolase C-A OS=*Danio rerio*	Q4KMC8	49	8.2	364	39.845
	Glyceraldehyde-3-phosphate dehydrogenase 2 OS=*Danio rerio*	Q5MJ86	42	6	335	36.084
	Fructose-bisphosphate aldolase A (Fragment) OS=*Thunnus albacares*	P86979	37	21.6	37	3.912
B1 (37 kDa)	Myosin heavy chain, fast skeletal muscle OS=*Cyprinus carpio*	Q90339	2858	24.9	1935	221.462
	Tropomyosin alpha-1 chain OS=*Liza aurata*	P84335	1494	81	284	32.710
	Tropomyosin alpha-1 chain OS=*Danio rerio*	P13104	1118	68	284	32.703
	Glyceraldehyde-3-phosphate dehydrogenase OS=*Danio rerio*	Q5XJ10	203	17.1	333	35.761
	Fructose-bisphosphate aldolase A OS=*Salmo salar*	B5DGM7	147	3.9	363	39.531
	Alpha-enolase OS=*Thunnus albacares*	I0J1J1	112	7.2	432	47.106
	Actin, alpha skeletal muscle OS=*Carassius auratus*	P49055	80	8.5	377	41.944

	Beta-enolase OS=*Salmo salar*	B5DGQ7	57	5.8	434	47.257
	Myosin light chain 1, skeletal muscle isoform OS=*Liza ramada*	P82159	31	5.9	186	200.54
	Fructose-bisphosphate aldolase A (Fragment) OS=*Thunnus albacares*	P86979	24	21.6	37	3.912
	Glyceraldehyde-3-phosphate dehydrogenase OS=*Oncorhynchus mykiss*	O42259	24	4.5	335	36.266
	Glyceraldehyde-3-phosphate dehydrogenase 2 OS=*Danio rerio*	Q5MJ86	24	6.9	335	36.084
C1 (242 kDa)	Myosin heavy chain, fast skeletal muscle OS=*Cyprinus carpio*	Q90339	25107	41.4	1935	221.462
	Actin, alpha skeletal muscle OS=*Carassius auratus*	P49055	735	58.4	377	41.944
	Glyceraldehyde-3-phosphate dehydrogenase OS=*Danio rerio*	Q5XJ10	645	24	333	35.761
	Beta-enolase OS=*Salmo salar*	B5DGQ7	324	24.7	434	47.257
	Fructose-bisphosphate aldolase A OS=*Salmo salar*	B5DGM7	280	22.3	363	39.531
	Myosin light chain 1, skeletal muscle isoform OS=*Liza ramada*	P82159	263	26.3	186	20.054
	Alpha-enolase OS=*Thunnus albacares*	I0J1J1	235	17.8	432	47.106

	Myosin light chain 3, skeletal muscle isoform OS=*Liza ramada*	P82160	231	34.5	148	16.358
	Tropomyosin alpha-1 chain OS=*Danio rerio*	P13104	54	23.6	284	32.703
	Glyceraldehyde-3-phosphate dehydrogenase 2 OS=*Danio rerio*	Q5MJ86	46	16.7	335	36.084
	Fructose-bisphosphate aldolase C-A OS=*Danio rerio*	Q4KMC8	36	12.6	364	39.845
C2 (90 kDa)	Myosin heavy chain, fast skeletal muscle OS=*Cyprinus carpio*	Q90339	11293	37.3	1935	221.462

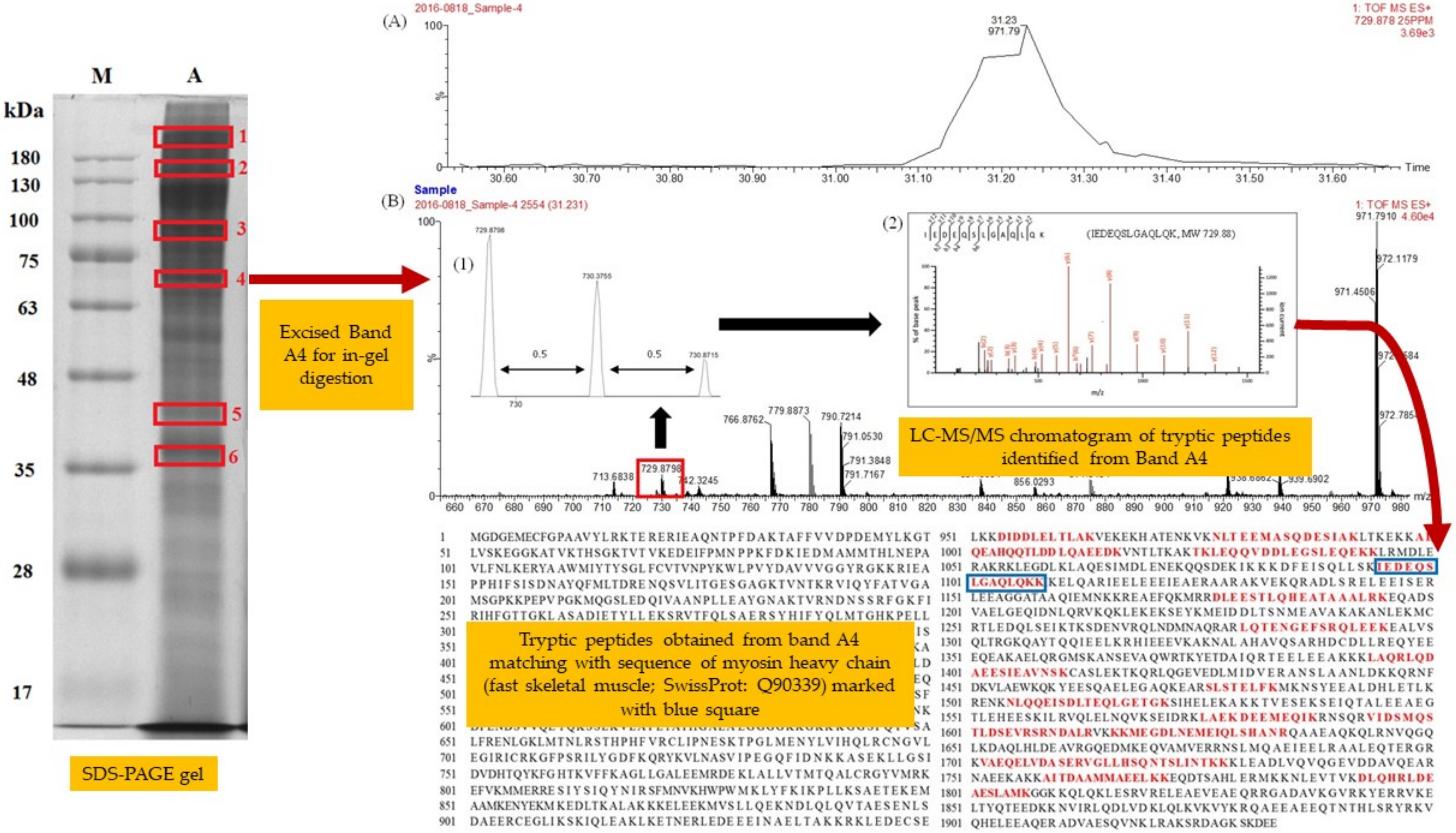

Figure S1. Corresponding tryptic peptides of mackerel steaming juice (MSJ) proteins from band A4 identified in myosin heavy chain (fast skeletal muscle) sequence
